# Surgical Aspects of Sleeve Gastrectomy Are Related to Weight Loss and Gastro-esophageal Reflux Symptoms

**DOI:** 10.1007/s11695-023-07018-y

**Published:** 2024-02-08

**Authors:** Hannu S. Lyyjynen, John R. Andersen, Ronald S. L. Liem, Tom Mala, Simon W. Nienhuijs, Johan Ottosson, Magnus Sundbom, Anders Thorell, Villy Våge

**Affiliations:** 1 Scandinavian Obesity Surgery Registry, Bergen, Norway; 2 Faculty of Health and Social Sciences, Western Norway University of Applied Sciences, Førde, Norway; 3 Centre of Health Research, Førde Hospital Trust, Førde, Norway; 4 Department of Surgery, Groene Hart Hospital, Gouda, Netherlands; 5 Nederlandse Obesitas Kliniek (NOK) in The Hague and Gouda, The Hague and Gouda, Netherlands; 6 Department of Gastrointestinal Surgery, Oslo University Hospital, Oslo, Norway; 7 Department of Surgery, Catharina Hospital, Eindhoven, Netherlands; 8 Department of Surgery, Örebro University Hospital, Örebro, Sweden; 9 Department of Surgical Sciences, Uppsala University, Uppsala, Sweden; 10 Karolinska Institutet, Department of Clinical Sciences, Danderyd Hospital, Stockholm, Sweden; 11 Department of Surgery and Anesthesia, Ersta Hospital, Stockholm, Sweden

**Keywords:** Sleeve gastrectomy, Bariatric surgery, Total weight loss, Gastro-esophageal reflux

## Abstract

**Introduction:**

A large variation in outcome has been reported after sleeve gastrectomy (SG) across countries and institutions. We aimed to evaluate the effect of surgical technique on total weight loss (TWL) and gastro-esophageal reflux disease (GERD).

**Methods:**

Observational cohort study based on data from the national registries for bariatric surgery in the Netherlands, Norway, and Sweden. A retrospective analysis of prospectively obtained data from surgeries during 2015–2017 was performed based on 2-year follow-up. GERD was defined as continuous use of acid-reducing medication. The relationship between TWL, *de novo* GERD and operation technical variables were analyzed with regression methods.

**Results:**

A total of 5927 patients were included. The average TWL was 25.6% in Sweden, 28.6% in the Netherlands, and 30.6% in Norway (*p* < 0.001 pairwise). Bougie size, distance from the resection line to the pylorus and the angle of His differed between hospitals. A minimized sleeve increased the expected total weight loss by 5–10 percentage points. Reducing the distance to the angle of His from 3 to just above 0 cm increased the risk of *de novo* GERD five-fold (from 3.5 to 17.8%).

**Conclusion:**

Smaller bougie size, a shorter distance to pylorus and to the angle of His were all associated with greater weight loss, whereas a shorter distance to angle of His was associated with more *de novo* reflux.

**Graphical Abstract:**

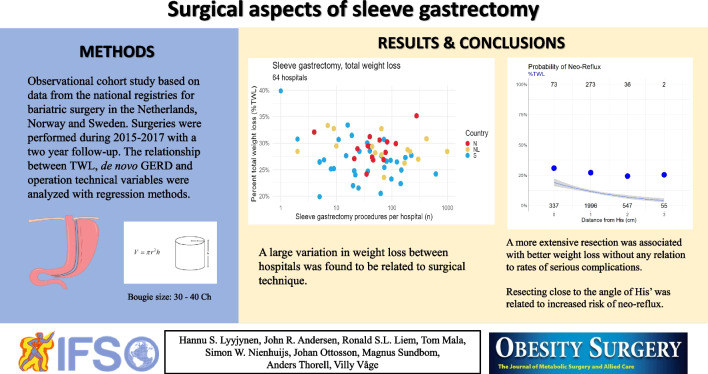

**Supplementary Information:**

The online version contains supplementary material available at 10.1007/s11695-023-07018-y.

## Introduction

The ultimate goal of bariatric surgery is to improve health and weight loss-achieved outcomes both in relation to obesity-related disorders and health-related quality of life (HRQL). However, the positive outcomes must be weighed against risks and potential adverse effects of the surgery. The weight loss obtained is dependent on the type of bariatric procedure performed but could also be due to variations in technical aspects of the specific procedure across countries, institutions, and surgeons.

We previously reported that the weight loss 1 year after Roux-en-Y gastric bypass was comparable between institutions in three European countries, with minor variations across institutions. However, we observed significant differences in weight loss after sleeve gastrectomy (SG) across the same institutions [1]. As these observations came from the same demographic, it could indicate an impact of variations in surgical technique with regard to the weight loss outcome post-SG. As SG is currently the most commonly performed bariatric procedure worldwide, optimizing the surgical technique is of major importance [2].

Gastro-esophageal reflux disease (GERD) is a potential side-effect of SG, and the reported prevalence of *de novo* GERD also differs between institutions [3]. Despite the extensive use of SG, few studies have focused on technical details in relation to weight loss and development of GERD. Although international consensus conferences provide guidelines on how to perform SG, these are mostly based on expert opinions rather than high level evidence [4, 5]. Based on data from three national registries, this study aimed to identify predictors of weight loss within 2 years after SG with special attention given to technical aspects of the procedure. We also explored potential associations between surgical technical aspects and new-onset use of acid reducing medication (ARM) as a proxy for *de novo* GERD. We hypothesized that a more extensive gastric resection would result in greater weight loss and also have an impact on the prevalence of GERD.

## Methods

This is an observational multinational cohort registry study. Retrospective analyses of prospectively obtained data from the national registers in Norway (SOReg-N), Sweden (SOReg-S), and the Netherlands (DATO) were carried out. Data from patients operated with primary SG from January 1, 2015 to December 31, 2017 were used. Surgical technical variables included bougie size measured in Charrière (Ch), the distance from pylorus to the starting point of the gastric resection (cm), and the distance from the angle of His to the gastric resection margin (cm).

### Data Sources

The Scandinavian Obesity Surgery Registry was established in 2007 in Sweden (SOReg-S) [6]. Norway joined in 2014 and received status as a national contributor to the SOReg in June 2015 (SOReg-N). All registry variables in Sweden and Norway apply the same definitions, and the database platform is identical. Patient inclusion to the registry is based on oral consent (“opt-out”) in Sweden and written consent (“opt-in”) in Norway. The capture rates during the study period when matched to the National Patient Registries were approximately ≈99% in Sweden and ≈70% in Norway. An identical system for auditing data to improve data quality has been developed and validated in both countries [7].

The Dutch Audit for Treatment of Obesity (DATO) started officially on January 1st 2015 [8]. Nationwide coverage is enforced by the Association of Surgeons of the Netherlands and inclusion is based on oral consent (“opt-out”). Reimbursement for a bariatric procedure is, however, only given for procedures registered in DATO, hence a capture rate of ≈100% is achieved. Validation is based on on-site visits followed by consecutive data-driven audits.

Although variables have an overlap of more than 90% between the SOReg and DATO, the definition for some of the variables differs. In these cases, only variables from SOReg were used.

### Inclusion Criteria

Patients undergoing primary SG performed with bougie size 28–40 Ch, distance from pylorus ≤ 6 cm, and distance from angle of His ≤ 3 cm were included. This aimed to exclude patients who underwent SG as the first part of a planned duodenal switch procedure. Distance from the angle of His is not a variable in DATO, and data from the Netherlands were therefore included if the two first criteria were fulfilled. The variables: education, smoking, depression, insulin use, musculoskeletal pain, and operation time were either missing or defined differently in the Dutch dataset and therefore not included. Only data from patients with a 2-year follow-up within a time span of 21–27 months (i.e., 640–820 days) was used in order to minimize a possible influence on weight loss due to different follow-up times between institutions.

### Clinical Practice

The National Institutes of Health Consensus Development Conference Statement for indication of bariatric surgery from 1991 in the presence of obesity-related diseases was practiced in all three countries although private clinics in Sweden also operated a number of patients with a lower BMI without such diseases [9]. Furthermore, patients with a BMI down to 30 kg/m^2^ and type 2 diabetes mellitus (T2DM) may have been offered metabolic surgery on an individual basis during parts of the study period [10]. Selection of SG as the preferred bariatric procedure was generally conducted at the discretion of the attending surgeon after shared decision-making between patients and providers. We have no information as to whether reflux symptoms or endoscopic findings such as hiatal hernia or esophagitis influenced the choice of procedure.

### Definitions and Outcome Measures

Obesity-related diseases were defined as ongoing use of medication (diabetes, hypertension, dyslipidemia, GERD, and depression) or continuous positive airway pressure (CPAP) in the case of sleep apnea. GERD was defined as daily use of ARM for the last 30 days and *de novo* GERD as use of ARM at 2 years only. The Clavien-Dindo Classification of Surgical Complications (CD) was used to categorize post-operative complications [11]. CD-grade IIIb or higher was classified as severe complications representing a need for surgical, endoscopic, and/or radiological intervention under general anesthesia.

Weight loss is presented as percent total weight loss after 2 years,

%*TWL* = (*W*_0_ − *W*_2_)/*W*_0_ × 100, where *W*_0_ is the weight at base registration and *W*_2_ at 2-year follow-up. We also calculated the percentage of patients reaching a minimum of 20% *TWL* for each center as a performance measure.

### Statistical Analysis

Data was analyzed according to intention-to-treat. A sensitivity analysis comparing pre-operative BMI and prevalence of GERD in the group with and without 2-year data was performed using the Mann-Whitney *U*-test. A potential statistical difference in baseline values between the three countries was explored pairwise with a Mann-Whitney *U*-test for continuous variables and a chi-squared test for proportions. A univariate linear regression analysis was performed to detect potential association on %*TWL* of any variable, whether pre-, peri- or post-operative. Due to lack of some data in the Dutch dataset, a multivariate analysis on all variables was performed on data from the SOReg registries only.

A possible relationship between surgical technique and weight loss was investigated with linear regression models, while relationship between surgical technique and *de novo* GERD was modeled with a logistic regression. Peri-operative predictors (bougie size, distance from pylorus, and distance from angle of His) with possibly quadratic effects were used to explain both outcome variables. The models were adjusted for age, sex, and either BMI or %*TWL* (in order to evaluate a potential effect of %*TWL* on GERD). Statistical significance for each coefficient was set at *p* < 0.05.

R version 4.2.1 was used in all analyses [12].

## Results

A total of 14,062 SG were identified, but 3262 of these were excluded due to missing data and another 262 because they were outside the inclusion criteria for operative technical specifications. Registry entries concerning follow-up data within norm time interval was available for 5927 of the remaining 10,538 patients (56.2%, Table 1). The initial mean BMI varied from 39.7 kg/m^2^ in Sweden to 44.0 kg/m^2^ in the Netherlands, with a mean BMI of 42.0 kg/m^2^ in the entire study group. In the sensitivity analysis the pre-operative BMI was 41.3 (*p* < 0.001) in the group without 2-year follow-up data and thus not included in the study. The prevalence of pre-operative GERD was 7.4% in the study group versus 6.6% in the group without 2-year follow-up data (*p* = 0.13). There were variations in bougie size and distance from the resection line to pylorus and the angle of His between hospitals and across countries. More severe complications were reported from the Netherlands (Table 1).

**Table 1:** Overview of pre-, peri-, and post-operative characteristics with comparison between countries (*n* = 5927)

Variable	Norway (N)	Sweden (S)	Netherlands (NL)	*p*-value
Pre-operative characteristics				
Number of procedures	*n* = 976	*n* = 2343	*n* = 2608	
Age years, mean (*SD*)	42.7 (10.8)	42.8 (10.9)	43.5 (11.9)	N-NL*, S-NL**
Female (%)	728 (74.6)	1883 (80.4)	1974 (75.7)	N-S***, S-NL***
BMI kg/m^2^, mean (*SD*)	42.2 (5.6)	39.7 (4.9)	44.0 (6.1)	All pairs ***
Education ≤ 10 years (%)	115/858 (13.4)	178/1506 (11.8)	-	0.29
Smoking (%)	166/976 (17.0)	330/2343 (14.1)	-	*
Depression (%)	126/976 (12.9)	334/2343 (14.3)	-	0.334
T2DM (%)	98/976 (10)	218/2343 (9.3)	293/2608 (11.2)	S-NL *
Data on insulin use	74/86	202/209	-	
Not using insulin	54/74	131/202	-	1
Using insulin	20/74	71/202	-	0.14
Musculoskeletal pain (%)	321/976 (32.9)	328/2342 (14.0)	-	***
GERD (%)	109/976 (11.2)	125/2343 (5.3)	202/2607 (7.7)	N-S,S-NL***,N-NL**
Sleep apnoea (%)	157/976 (16.1)	203/2343 (8.7)	253/2608 (9.7)	N-S, N-NL***, S-NL 0.3
Weight loss kg, mean (*SD*)	5.5 (6)	5.7 (4.7)	4.1 (6.6)	N-NL,S-NL ***
Perioperative characteristics				
Bougie size Ch, mean (*SD*)	33.4 (2.1)	34.9 (1.1)	35.2 (2.3)	All pairs ***
Distance pylorus cm, mean (*SD*)	3.6 (1.5)	4.6 (0.7)	4.9 (0.9)	All pairs ***
Distance His cm, mean (*SD*)	0.8 (0.6)	1.2 (0.6)	-	***
Operation time min, mean (*SD*)	63.4 (31.5)	48 (17.8)	-	***
Post-operative characteristics				
Length of stay days, mean	2.1	1.5	2.0	All pairs***
Complications *CD* ≥ 3b, day [0–30], (%)	15 (1.5)	36 (1.6)	93 (3.6)	N-NL**, S-NL***

Significant *p*-values *** < 0.001 ** < 0.01 * < 0.05

*BMI* body mass index, *T2DM* type 2 diabetes mellitus, *CD* Clavien-Dindo

### Weight Loss and Predictors of Weight Loss

**Fig. 1:**
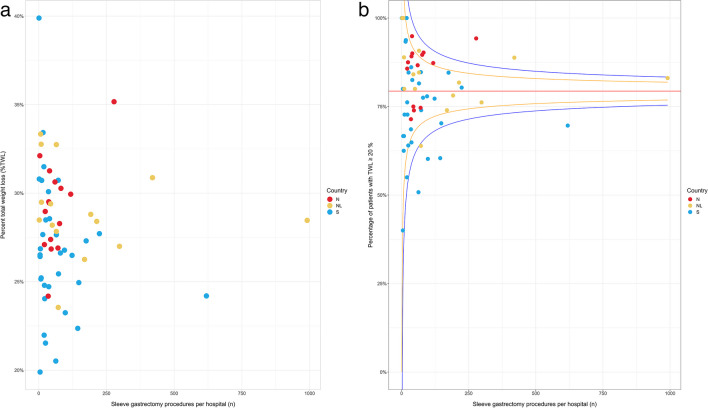
**a**. The average percentage of total weight loss (%TWL) two years after surgery reported per hospital (*n* = 64). **b**. The percentage of patients registered with a total weight loss ≥ 20 % two years after surgery reported by hospital

The mean %*TWL* varied from 20 to 40% across institutions (Fig. 1a). There was also a large variation in the percentage of patients who reached a %*TWL* ≥ 20 (Fig. 1b). On average, 79.3% (range 40.0 to 100.0%) reached this cut-off value. In the univariate analysis of pre-operative characteristics, female sex, higher BMI, and smoking were associated with higher total weight loss, while higher age, receiving treatment for depression, T2DM, reflux and sleep apnea were associated with lower weight loss (Supplementary Table 1). For intra-operative variables, a smaller bougie size, a shorter distance to pylorus and the angle of His, and a longer operating time were all associated with higher weight loss. In the multivariate analysis (data only from Norway and Sweden), female sex, higher BMI, smoking, pre-operative weight loss, smaller bougie size, shorter distance to pylorus, shorter distance to angle of His, and longer hospital stays were all associated with higher weight loss, while higher age, depression, and T2DM were associated with lower weight loss (Table 2).

**Table 2:** Predictors of %*TWL* in a multivariate analysis (Norway and Sweden, *n* = 3319), adj. *R*-squared: 0.22

Variable	Cases / *n* with available data (%)	Slope	*Se*	*p*-value
Pre-operative				
Age (year)	/3319 (100)	−0.14	0.018	< 0.001
Female	2611/3319 (78.7)	2.08	0.48	< 0.001
BMI (kg/m^2^)	/3319 (100)	0.26	0.04	< 0.001
Education ≤ 10 years	293/2364 (12.4)	−0.648	0.56	0.24
Smoking	496/3319 (14.9)	2.19	0.49	< 0.001
Depression	460/3319 (13.9)	−1.55	0.54	0.0043
T2DM	316/3319 (9.5)			
Data on insulin use	276/295 (93.6)			
Not using insulin vs no T2DM	185/276 (67.0)	−3.57	0.80	< 0.001
Using insulin vs no T2DM	91/276 (33.0)	−4.97	1.15	< 0.001
GERD	234/3319 (7.1)	−0.98	0.72	0.17
Sleep apnoea	360/3319 (10.8)	−0.93	0.64	0.14
Weight change (kg)	/3186 (100)	0.35	0.04	< 0.001
Peri-operative				
Bougie size (Ch)	/3319 (100)	−0.93	0.14	< 0.001
Distance, pylorus (cm)	/3319 (100)	−1.14	0.20	< 0.001
Distance, His angle (cm)	/3319 (100)	−1.51	0.32	< 0.001
Operation time (min)	/3319 (100)	0.0054	0.008	0.53
Postoperative				
Length of stay (days)	/3319 (100)	0.259	0.112	0.02
Complication *CD* ≥ 3b, day [0–30]	51/3305 (1.5)	1.21	1.55	0.44

*BMI* body mass index, *T2DM* type 2 diabetes mellitus, *CD* Clavien-Dindo, *Slope* regression coefficient, *Se* standard error

**Fig. 2:**
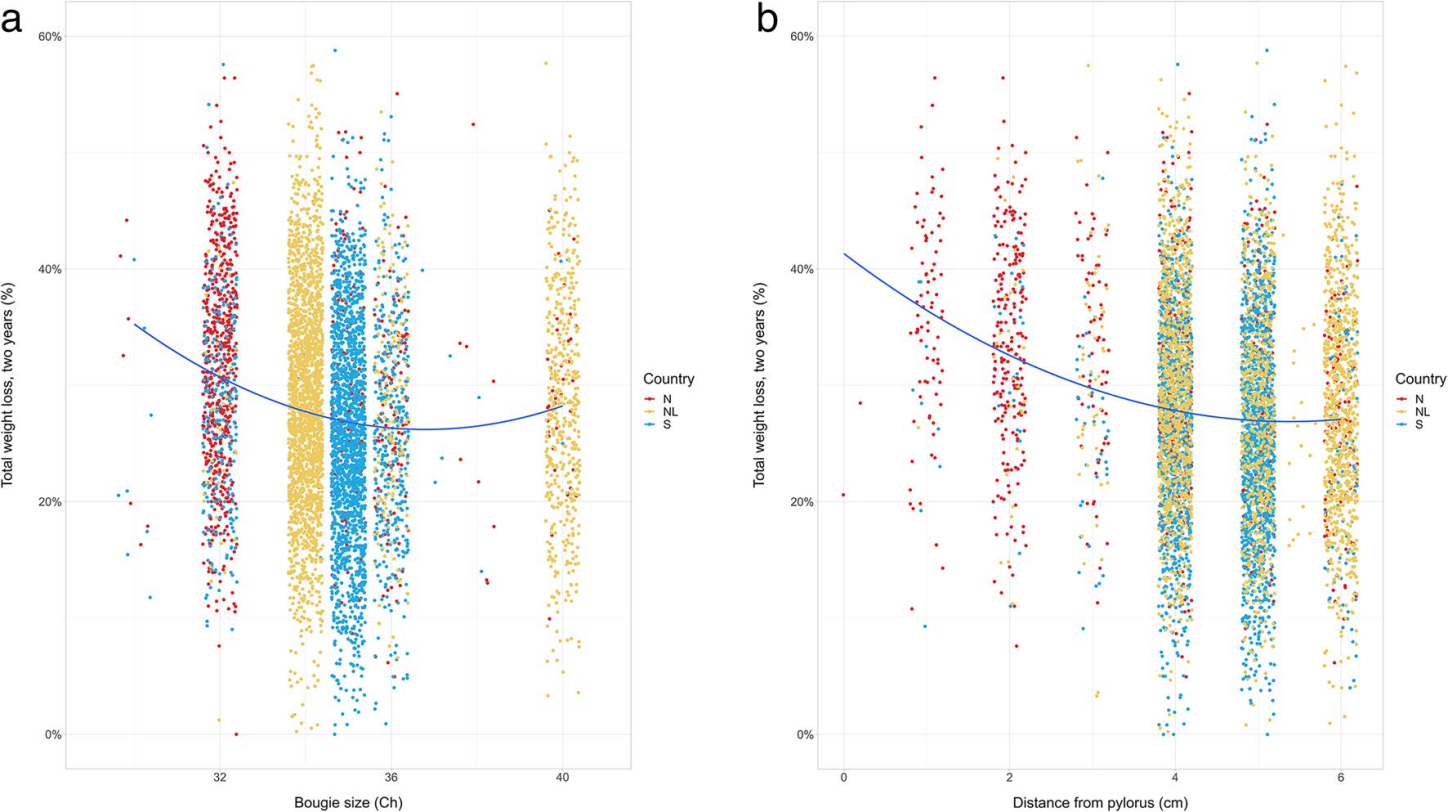
**a**. Percent total weight loss (%TWL) versus  bougie size. **b**. Percent total weight loss (%TWL) versus distance from pylorus

When evaluating how the surgical technical variables separately influenced weight loss, we found a linear relationship between the %*TWL* and the distance to angle of His (only SOReg data available), whereas for bougie size and distance to pylorus, there was a quadratic relation (i.e., the smaller the bougie size or distance to pylorus, the steeper the increase in %*TWL*; Supplementary Table 2). This effect is visualized in Fig. 2a and b, where a smaller bougie size and a shorter distance to pylorus both were associated with a larger %*TWL* at 2 years in a quadratic manner.

### Reflux and Predictors of Change in Reflux Status

A total of 436 patients (7.5%) were treated for GERD pre-operatively while 543 (9.3%) received treatment for GERD 2 years after the operation. While 282 of the 436 (65%) obtained GERD remission, 419 out of 5382 (7.8%) had de novo GERD at 2 years. Statistically significant predictors of GERD remission were higher age and a shorter distance to pylorus (Supplementary Table 3). Greater weight loss was not associated with GERD remission.

**Fig. 3:**
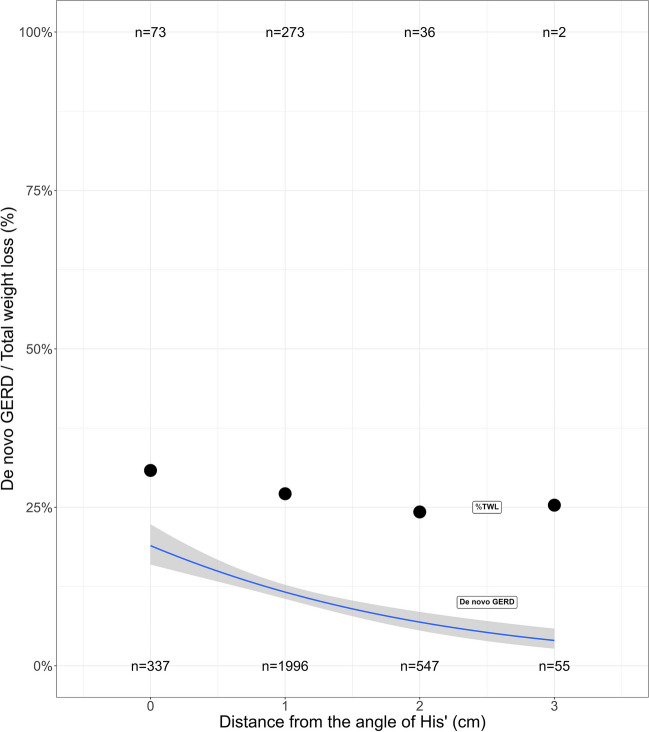
*De novo* GERD and percent total weight loss (%TWL) versus distance from His. The curve in the figure is a logistic regression model for the empirical probability of developing *de novo* GERD after SG. The points represent the total weight loss. The number of patients with *de* *novo* GERD are presented at the top of the grid, while the number of patients without *de* *novo* GERD are presented  at the bottom of the grid

The occurrence of *de novo* GERD was significantly influenced by a shorter distance to the angle of His (Supplementary Table 4). Bougie size was of borderline significance for the development of reflux when %*TWL* at 2 years was included as a covariate in the final model (*p* = 0.04-0.05). The smaller the distance to angle of His, the larger the occurrence of *de novo* GERD. As confirmed by the logistic regression model, the empirical probability increased with over 10 percentage points up to ≈18% as the distance was reduced from 3 to 0 cm (Fig. 3).

### Complications

Overall, 144 patients (2.4%) were registered with a serious complication (*CD* ≥ IIIb) within the first 30 days and another 90 patients (1.6%) had a serious complication between 31 and 820 days after surgery. We found no relationship between serious complications and any value of the operative variables bougie size, distance to pylorus, and distance to angle of His (data not shown).

## Discussion

Based on data from 5927 patients prospectively included in SOReg and DATO, we explored possible predictors for weight loss and development of *de novo* GERD 2 years after SG with a particular focus on operative technique. Bougie size, distance from pylorus, and distance to the angle of His varied between countries and institutions. The mean *TWL* in Norway was 30.6%, Sweden 25.6%, and the Netherlands 28.6% (*p* < 0.001), with large variations between hospitals. The incidence of *de novo* GERD was 7.8%. A smaller bougie size, shorter distance to pylorus and to the angle of His were all and independently highly related to a higher %*TWL*. A shorter distance to angle of His was also associated with *de novo* GERD.

A more extensive resection as indicated by the use of a smaller bougie size, a shorter distance to pylorus and to the angle of His were all associated with higher weight loss without any significant difference in serious complications (*CD* ≥ 3b). Similar conclusions have been drawn from several studies on antral resections and bougie size [13–15]. Although available data suggests that serious complications can be kept to a minimum also with a small bougie size, an extensive resection could increase the need for intravenous fluids in the early post-operative period which to some extent can explain our finding of a longer hospital stay among patients with more extensive resection [16].

We observed that a shorter distance to angle of His was associated with better weight loss as well as an increased risk of developing GERD. Weight loss is generally recommended in the treatment of reflux; however, greater weight loss was not related to GERD remission in this series. Stapling close to the angle of His could increase the pressure in the lower esophageal sphincter (LES), as reported by Petersen et al, but at the same time, dissection and stapling at and around the hiatus might cause breakdown of anatomical structures like the phreno-esophageal membrane which has an important role in keeping the LES intra-abdominally [17]. Hence, resecting close to the angle of His could increase the likelihood of an axial separation between the diaphragmatic crura and the gastro-esophageal junction, allowing intra-thoracic migration of the gastric remnant over time [18, 19]. On the other hand, adequate dissection and exposure of the proximal part of the stomach are important to avoid misidentification of the angle of His and a retained fundus [20]. It is considered crucial that the LES remains in an intra-abdominal position and in close proximity to the diaphragmatic crura in order to prevent reflux [21]. This leaves the surgeon with a delicate balance between performing an adequate exposure and resection of the fundus while at the same time not causing an anatomical aberration that might allow intra-thoracic migration of the gastric remnant. Anti-reflux procedures were not combined with sleeve at the time, and other techniques such as crural repair or gastropexy were only reported in low numbers and not standardized, making adequate statistical evaluation of the influence of these measures impossible. We have no clear explanation for the higher GERD remission rate with higher age, but we could speculate that the esophageal mucosa becomes less sensitive over time [22, 23].

Strengths of this study include the large and unselected study sample with sub-samples from multiple centers in three European countries and with variations in the surgical technique. Data was collected prospectively, and the use of %*TWL* as the outcome for weight loss should alleviate a potential influence of pre-operative differences in BMI between patients and cohorts [24, 25]. National quality registers with high rates for acquisition, validity, and follow-up are powerful tools for evaluating treatment outcomes [26]. International collaboration including data from such registries using equally defined variables enables exploration of how technical variations in the performance of a procedure affect outcome based on large sample sizes [27]. This is knowledge that would not be possible to achieve otherwise [28]. Furthermore, results can be compared between institutions both on a national and an international level and weight-loss-related outcomes after SG such as remission rates for T2DM should be considered related to the actual weight loss performance for the actual hospital [29].

Limitations with our study include the observational design which opens for residual confounding and restricts the ability to draw causal inferences. For example, all Norwegian procedures were publicly financed and all Dutch procedures insurance-paid, while in Sweden about 25% were self-financed. A potential influence from differences in pre- and post-operative programs between hospitals could not be assessed, neither could a potential difference in the surgical performance like the assistant’s force of traction on the specimen during the resection, since this is not quantified in the registries. Furthermore, we have no information on how the distances from pylorus or the angle of His to the resection line was measured.

Due to differences in the capture rate and data availability across national registries, all patients could not be included in the final analysis. Our sensitivity analysis showed that pre-operative BMI was statistically lower in patients without follow-up than in patients in the study group. However, since this difference was small and the prevalence of GERD was very similar, we assume that there should be a low risk of selection bias related to missing follow-up data. The use of ARM as a proxy for GERD confers some limitations including potential different practices for prescription of such medications, particularly across nations. As the prevalence of GERD was not objectively evaluated by endoscopy or pH-metry, sub-clinical or asymptomatic GERD may not have been revealed.

Future research is encouraged to explore effectful surgical techniques with low risks, e.g., gastropexy (suturing the divided omentum to the gastric remnant) that may keep the LES intra-abdominaly in order to reduce the risk of reflux [30]. Furthermore, the balance between the size and shape of the gastric remnant and HRQL, including the ability to enjoy food of various types, needs to be studied.

In conclusion, the surgical techniques applied and the *TWL* varied between institutions and countries. A smaller bougie size, shorter distance from the staple line to pylorus, and to the angle of His were independently related to increased weight loss. As a shorter distance between the proximal staple line and angle of His also was associated with increased risk of *de novo* GERD, the surgeon faces a delicate balance between achieving superior weight loss and at the same time avoiding the onset of reflux.

### Supplementary Information

ESM 1 (DOCX 21 kb)

ESM 2 (DOCX 19 kb)

ESM 3 (DOCX 20 kb)

ESM 4 (DOCX 25 kb)

## Data Availability

Data will be made available on reasonable request for scientific collaborations after the execution of appropriate data sharing agreements.

## Declarations

### Disclaimer

Original article. The main results have been presented at ZOOM Forward 22 (4 May 2022), the joint congress on obesity of the European Association of the Study of Obesity and the International Federation for the Surgery of Obesity and metabolic disorders-European Chapter.
