# Supplementary material for: Surgical Aspects of Sleeve Gastrectomy Are Related to Weight Loss and Gastro-esophageal Reflux Symptoms
Source: Obes Surg. 2024 Feb 8;34(3):902–10. doi: 10.1007/s11695-023-07018-y (PMC10899332; doi:10.1007/s11695-023-07018-y)
Supplement: Supplementary file 1 — (DOCX 21 kb) [file 11695_2023_7018_MOESM1_ESM.docx]

| **Supplementary TABLE 1. Predictors of %TWL in a univariate analysis (n=5927). Example: One unit higher BMI leads to 0.32% better TWL.** | | | | |
| --- | --- | --- | --- | --- |
| **Variable** | **Cases / n with**  **available data (%)** | **Slope** | **Se** | **P-value** |
| **Preoperative** |  |  |  |  |
| Age (yr) | /5927 (100) | -0.17 | 0.011 | < 0.001 |
| Female | 4585/5927 (77.4) | 1.45 | 0.30 | < 0.001 |
| BMI (kg/m^2^) | /5927 (100) | 0.32 | 0.021 | < 0.001 |
| Education ≤ 10 yrs | 293/2364 (12.4) | -0.99 | 0.61 | 0.10 |
| Smoking | 496/3319 (14.9) | 2.41 | 0.47 | < 0.001 |
| Depression | 460/3319 (13.9) | -1.91 | 0.49 | < 0.001 |
| T2DM | 609/5927 (10.3) | -4.74 | 0.41 | < 0.001 |
| Data on insulin use | 276/295 (93.6) |  |  |  |
| Not using insulin | 185/276 (67.0) | -4.49 | 0.73 | <0.001 |
| Using insulin | 91/276 (33.0) | -5.15 | 0.93 | < 0.001 |
| Musculo-sceletal pain | 649/3318 (19.6) | 0.54 | 0.42 | 0.20 |
| GERD | 436/5926 (7.4) | -1.21 | 0.49 | < 0.012 |
| Sleep apnoea | 613/5927 (10.3) | -1.90 | 0.41 | < 0.001 |
| Weight change (kg) | /4348 (100) | 0.28 | 0.026 | < 0.001 |
| **Perioperative** |  |  |  |  |
| Bougie size (Ch) | /5927 (100) | -0.34 | 0.063 | < 0.001 |
| Distance, pylorus (cm) | /5927 (100) | -1.31 | 0.12 | < 0.001 |
| Distance, His’ angle (cm) | /3319 (100) | -2.81 | 0.28 | < 0.001 |
| Operation time (min) | /3319 (100) | 0.040 | 0.0071 | < 0.001 |
| **Postoperative** |  |  |  |  |
| Length of stay (d) | /5926 (100) | 0.012 | 0.0088 | 0.18 |
| Complication CD ≥3b, day [0-30] | 144/5888 (2.4) | 0.77 | 1.0 | 0.44 |
| Abbreviation: BMI, body mass index; T2DM, type 2 diabetes mellitus; CD, Clavien-Dindo; Slope, Regression coefficient; Se, Standard error | | | | |
