# Supplementary material for: Surgical Aspects of Sleeve Gastrectomy Are Related to Weight Loss and Gastro-esophageal Reflux Symptoms
Source: Obes Surg. 2024 Feb 8;34(3):902–10. doi: 10.1007/s11695-023-07018-y (PMC10899332; doi:10.1007/s11695-023-07018-y)
Supplement: Supplementary file 2 — (DOCX 19 kb) [file 11695_2023_7018_MOESM2_ESM.docx]

| **Supplementary Table 2.** Total weight loss models for five different cohorts. The effect of bougie size, distance to pylorus and distance to the angle of His’ on percent total weight loss adjusted for preoperative age, sex and BMI. A quadratic approach is used to highlight possible non-linear effects. | | | | | | | | | | |
| --- | --- | --- | --- | --- | --- | --- | --- | --- | --- | --- |
|  | Norway (N) |  | Sweden (S) |  | Netherlands (NL) |  | N+S |  | N+S+NL |  |
| n | 976 |  | 2343 |  | 2608 |  | 3319 |  | 5927 |  |
|  | Estimate | P-value | Estimate | P-value | Estimate | P-value | Estimate | P-value | Estimate | P-value |
| (Intercept) | 177.08 | 0.003 | 300.26 | 0.003 | -150.48 | 0.10 | 274.44 | <0.001 | **171.59** | <0.001 |
| Age | -0.16 | <0.001 | -0.16 | <0.001 | -0.14 | <0.001 | -0.16 | <0.001 | **-0.15** | <0.001 |
| Female | 0.59 | 0.36 | 2.02 | <0.001 | 1.21 | 0.005 | 1.51 | <0.001 | **1.27** | <0.001 |
| BMI | 0.34 | <0.001 | 0.30 | <0.001 | 0.18 | <0.001 | 0.33 | <0.001 | **0.29** | <0.001 |
| Bougie size | -8.18 | 0.02 | -15.43 | 0.01 | 10.37 | 0.04 | -13.79 | <0.001 | **-7.58** | <0.001 |
| (Bougie size)^2^ | 0.11 | 0.03 | 0.22 | 0.01 | -0.14 | 0.04 | 0.19 | <0.001 | **0.10** | <0.001 |
| Distance from pylorus | -1.73 | 0.06 | -2.72 | 0.10 | -4.18 | 0.02 | -2.25 | 0.002 | **-3.85** | <0.001 |
| (Distance from pylorus)^2^ | 0.12 | 0.37 | 0.21 | 0.27 | 0.36 | 0.06 | 0.16 | 0.01 | **0.35** | <0.001 |
| Distance from His’ angle | -3.40 | 0.002 | -1.29 | 0.21 |  |  | -2.51 | <0.001 |  |  |
| (Distance from His’ angle)^2^ | 1.20 | 0.005 | -0.22 | 0.55 |  |  | 0.35 | 0.18 |  |  |
| r.squared | 0.17 |  | 0.10 |  | 0.06 |  | 0.16 |  |  |  |
| adj.r.squared | 0.17 |  | 0.09 |  | 0.05 |  | 0.16 |  |  |  |
