# Supplementary material for: Surgical Aspects of Sleeve Gastrectomy Are Related to Weight Loss and Gastro-esophageal Reflux Symptoms
Source: Obes Surg. 2024 Feb 8;34(3):902–10. doi: 10.1007/s11695-023-07018-y (PMC10899332; doi:10.1007/s11695-023-07018-y)
Supplement: Supplementary file 3 — (DOCX 20 kb) [file 11695_2023_7018_MOESM3_ESM.docx]

| **Supplementary Table 3.** Four models for potential GERD remission (Norway and Sweden). The effect of bougie size, distance to pylorus and distance to the angle of His’ on GERD remission, adjusted for preoperative age, sex, BMI or percent total weight loss. A quadratic approach is used to highlight possible non-linear effects. | | | | | | | | |  |  |  |  |  |  |  |
| --- | --- | --- | --- | --- | --- | --- | --- | --- | --- | --- | --- | --- | --- | --- | --- |
|  | Norway |  | Sweden |  | Both |  | Both |  |  |  |  |  |  |  |  |
| n | 976 |  | 2343 |  | 3319 |  | 3319 |  |  |  |  |  |  |  |  |
| Predictors | Estimate | P-value | Estimate | P-value | Estimate | P-value | Estimate | P-value |  |  |  |  |  |  |  |
| (Intercept) | -0.54 | 0.74 | 2.64 | 0.13 | 0.14 | 0.88 | 0.17 | 0.85 |  |  |  |  |  |  |  |
| Age | 0.000097 | 0.89 | 0.0013 | <0.001 | 0.00097 | 0.00101 | 0.0009 | 0.0015 |  |  |  |  |  |  |  |
| Female | -0.047 | 0.007 | 0.0066 | 0.42 | -0.011 | 0.17 | -0.010 | 0.17 |  |  |  |  |  |  |  |
| BMI |  |  |  |  | -0.00001 | 0.98 |  |  |  |  |  |  |  |  |  |
| Year 2, TWL | 0.0002 | 0.81 | -0.0003 | 0.36 |  |  | -0.00011 | 0.74 |  |  |  |  |  |  |  |
| Distance, His’ angle | -0.041 | 0.15 | 0.0088 | 0.62 | -0.023 | 0.11 | -0.023 | 0.11 |  |  |  |  |  |  |  |
| (Distance, His’ angle)^2^ | 0.009 | 0.42 | -0.0003 | 0.96 | 0.0087 | 0.10 | 0.0088 | 0.10 |  |  |  |  |  |  |  |
| Distance, pylorus | -0.04 | 0.11 | -0.029 | 0.31 | -0.061 | <0.001 | -0.061 | <0.001 |  |  |  |  |  |  |  |
| (Distance, pylorus)^2^ | 0.0029 | 0.4 | 0.0035 | 0.30 | 0.0061 | 0.002 | 0.0061 | 0.002 |  |  |  |  |  |  |  |
| Bougie size | 0.045 | 0.63 | -0.16 | 0.13 | 0.41 | 0.94 | 0.0024 | 0.96 |  |  |  |  |  |  |  |
| (Bougie size)^2^ | -0.00068 | 0.61 | 0.0023 | 0.13 | -0.0001 | 0.89 | -0.00008 | 0.92 |  |  |  |  |  |  |  |
| r.squared | 0.039 |  | 0.012 |  | 0.023 |  | 0.023 |  |  |  |  |  |  |  |  |
| adj.r.squared | 0.030 |  | 0.008 |  | 0.020 |  | 0.020 |  |  |  |  |  |  |  |  |
