# Supplementary material for: Surgical Aspects of Sleeve Gastrectomy Are Related to Weight Loss and Gastro-esophageal Reflux Symptoms
Source: Obes Surg. 2024 Feb 8;34(3):902–10. doi: 10.1007/s11695-023-07018-y (PMC10899332; doi:10.1007/s11695-023-07018-y)
Supplement: Supplementary file 4 — (DOCX 25 kb) [file 11695_2023_7018_MOESM4_ESM.docx]

| **Supplementary Table 4.** Four models for potential de novo GERD (Norway and Sweden). The effect of bougie size, distance to pylorus and distance to the angle of His’ on de novo GERD, adjusted for preoperative age, sex, BMI or percent total weight loss. A quadratic approach is used to highlight possible non-linear effects. | | | | | | | | |  |  |  |  |  |  |  |
| --- | --- | --- | --- | --- | --- | --- | --- | --- | --- | --- | --- | --- | --- | --- | --- |
|  | Norway |  | Sweden |  | Both |  | Both |  |  |  |  |  |  |  |  |
| n | 976 |  | 2343 |  | 3319 |  | 3319 |  |  |  |  |  |  |  |  |
| Predictors | Estimate | P-value | Estimate | P-value | Estimate | P-value | Estimate | P-value |  |  |  |  |  |  |  |
| (Intercept) | -3.96 | 0.09 | -0.31 | 0.93 | -3.11 | 0.05 | -2.98 | 0.06 |  |  |  |  |  |  |  |
| Age | 0.0019 | 0.06 | 0.0012 | 0.05 | 0.0014 | <0.001 | 0.0014 | 0.009 |  |  |  |  |  |  |  |
| Female | 0.052 | 0.037 | 0.054 | <0.001 | 0.055 | <0.001 | 0.054 | <0.001 |  |  |  |  |  |  |  |
| BMI |  |  |  |  | 0.00088 | 0.42 |  |  |  |  |  |  |  |  |  |
| Year 2, TWL | -0.0029 | 0.019 | 0.0011 | 0.13 |  |  | 0.000027 | 0.97 |  |  |  |  |  |  |  |
| Distance, His’ angle | -0.063 | 0.13 | -0.051 | 0.15 | -0.064 | 0.011 | -0.065 | 0.01 |  |  |  |  |  |  |  |
| (Distance, His’ angle)^2^ | -0.00045 | 0.98 | 0.0014 | 0.91 | 0.0037 | 0.69 | 0.0040 | 0.67 |  |  |  |  |  |  |  |
| Distance, pylorus | -0.0015 | 0.97 | -0.045 | 0.43 | -0.033 | 0.20 | -0.035 | 0.19 |  |  |  |  |  |  |  |
| (Distance, pylorus)^2^ | -0.0012 | 0.81 | 0.0073 | 0.27 | 0.0047 | 0.17 | 0.0048 | 0.17 |  |  |  |  |  |  |  |
| Bougie size | 0.24 | 0.075 | 0.018 | 0.93 | 0.19 | 0.043 | 0.18 | 0.05 |  |  |  |  |  |  |  |
| (Bougie size)^2^ | -0.0034 | 0.072 | -0.00019 | 0.95 | -0.0028 | 0.042 | -0.0027 | 0.049 |  |  |  |  |  |  |  |
| r.squared | 0.036 |  | 0.016 |  | 0.019 |  | 0.018 |  |  |  |  |  |  |  |  |
| adj.r.squared | 0.027 |  | 0.012 |  | 0.016 |  | 0.016 |  |  |  |  |  |  |  |  |
